# Predicting Pedestrian Flow: A Methodology and a Proof of Concept Based on Real-Life Data

**DOI:** 10.1371/journal.pone.0083355

**Published:** 2013-12-27

**Authors:** Maria Davidich, Gerta Köster

**Affiliations:** 1 Corporate Technology, Siemens AG, Munich, Bavaria, Germany; 2 Department of Computer Science, Munich University of Applied Sciences, Munich, Bavaria, Germany; University of Warwick, United Kingdom

## Abstract

Building a reliable predictive model of pedestrian motion is very challenging: Ideally, such models should be based on observations made in both controlled experiments and in real-world environments. De facto, models are rarely based on real-world observations due to the lack of available data; instead, they are largely based on intuition and, at best, literature values and laboratory experiments. Such an approach is insufficient for reliable simulations of complex real-life scenarios: For instance, our analysis of pedestrian motion under natural conditions at a major German railway station reveals that the values for free-flow velocities and the flow-density relationship differ significantly from widely used literature values. It is thus necessary to calibrate and validate the model against relevant real-life data to make it capable of reproducing and predicting real-life scenarios. In this work we aim at constructing such realistic pedestrian stream simulation. Based on the analysis of real-life data, we present a methodology that identifies key parameters and interdependencies that enable us to properly calibrate the model. The success of the approach is demonstrated for a benchmark model, a cellular automaton. We show that the proposed approach significantly improves the reliability of the simulation and hence the potential prediction accuracy. The simulation is validated by comparing the local density evolution of the measured data to that of the simulated data. We find that for our model the most sensitive parameters are: the source-target distribution of the pedestrian trajectories, the schedule of pedestrian appearances in the scenario and the mean free-flow velocity. Our results emphasize the need for real-life data extraction and analysis to enable predictive simulations.

## Introduction

An unbroken trend to urbanization and a growing taste for mass events are among the many reasons why we experience very dense crowds more and more often. The challenge is to ensure safety and comfort for people in such situations. Pedestrian stream simulations are nowadays considered a means to mitigate risk in dense crowds. Buildings and events can be planned with a focus on safety, making use of virtual experience gained from pedestrian streams simulations [1]–[7]. With the help of a simulation tool, a trained user can run through multiple “what-if” scenarios to gain experience for situations where it is impossible, uneconomical or even unethical to gather real experience [8], [9]. Moreover, since recently pedestrian stream models aim not only at simulating “what-if” scenarios, but also at short term prediction of real-life scenarios. Short term simulation predictions could be used to warn of critical situations such as danger of high densities.

Pedestrian stream simulations are advantageous only if capable of correctly reproducing pedestrian motion. Ideally, the models for social or natural phenomena should be developed on the basis of observations gathered via controlled experiments and field studies. As soon as the basic model is constructed, one should ensure that it reflects the real-life scenario being simulated. An accurate reproduction of known data, without being an exact proof, would indicate that the simulator may be trusted for collecting virtual experience or for realistic predictions. To transform a basic model into a model capable of reproducing real-life scenarios, it is thus necessary to calibrate and validate the model against the relevant real-life data.

A number of pedestrian models have been proposed ranging from macroscopic to microscopic simulations [10]. However, construction of reliable models so far has been hindered by a lack of sufficient data from real-life scenarios. Pedestrian motion models neither always come straight from observations nor are rigorously checked against them. Instead most models are calibrated and validated only against literature values or laboratory experiments [11]. They usually focus on a specific phenomenon such as: deceleration with increasing density according to a given fundamental diagram [12], lane formations in bi-directional pedestrian flows [13]–[16], pedestrian dynamics near a bottleneck [17], waiting zones [18], oscillations of flow-direction at doors [19] or dynamics within highly dense crowds in [20]. In the very best case models are calibrated and validated against relatively small and simple real-life scenarios [21] which in most cases again represent some single, isolated phenomenon. The very few examples of simulation calibration against real-life data include scenarios focused on entrances into escalators [22], queuing at a train station [21], bottleneck gates at a train station [23] and bi-directional flow in a corridor [24], [25]. Such calibration approach is very helpful and fully justified when one seeks to better understand an isolated phenomenon. However, one cannot expect that calibration solely based on an isolated phenomenon would be sufficient to reproduce a complex real-life scenario. Here, a more holistic attempt is necessary. The authors are not aware of any publications on comprehensive calibration.

In this paper we present a method for calibrating a cellular automata-based simulation against a complex real-life scenario observed at a major German railway station. Based on this real-life scenario we also examine the applicability of standard literature suggestions for free-flow velocity values and fundamental diagrams. We calibrate our simulation against pedestrian motion parameters extracted from video data. The success of the proposed approach is demonstrated by validation of our calibrated simulation against the observed real-life scenario. The validation is performed by comparing an aggregated quantity – pedestrian density evolution in the area of observation.

This paper is organized as follows: The Results section starts with an *Scenario and observational parameters: a German railway station* subsection on observations at a major German railway station. An overview of assumptions and restrictions of the benchmark model is given in *Simulation tool*. In *Methodology* we identify parameters critical for calibrating simulations and suggest how to feed them into a simulation model. Then the calibrated benchmark simulation is compared with measurements in *Proof of concept: Validation and sensitivity study*. The Discussion part summarizes the results. At the end the *Materials and methods* section gives insight into the video tracking technology we used.

## Results

### Scenario and observational parameters: a German railway station

**Figure 1:**
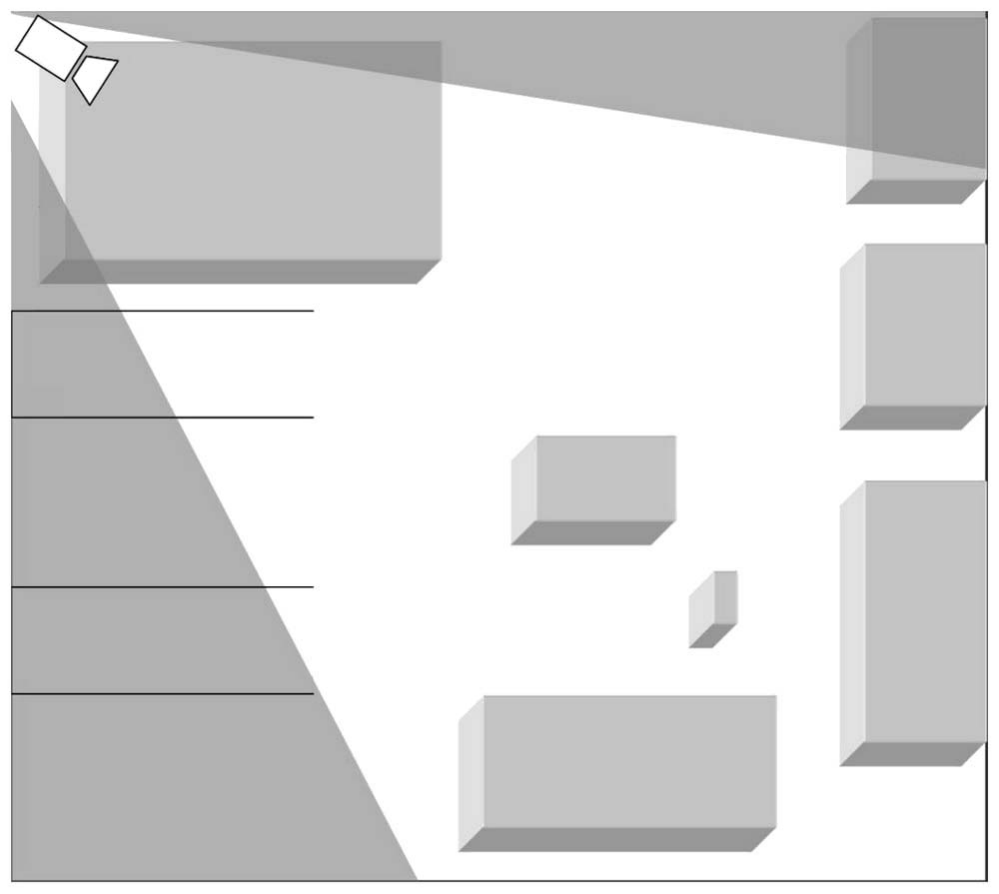
Schematic representation of the measurement experiment: White area corresponds to the vision field camera mounted in the upper left corner of the figure. There are platforms on the left side, and obstacles, such as food stalls, in the middle and on the right side.

**Figure 2:**
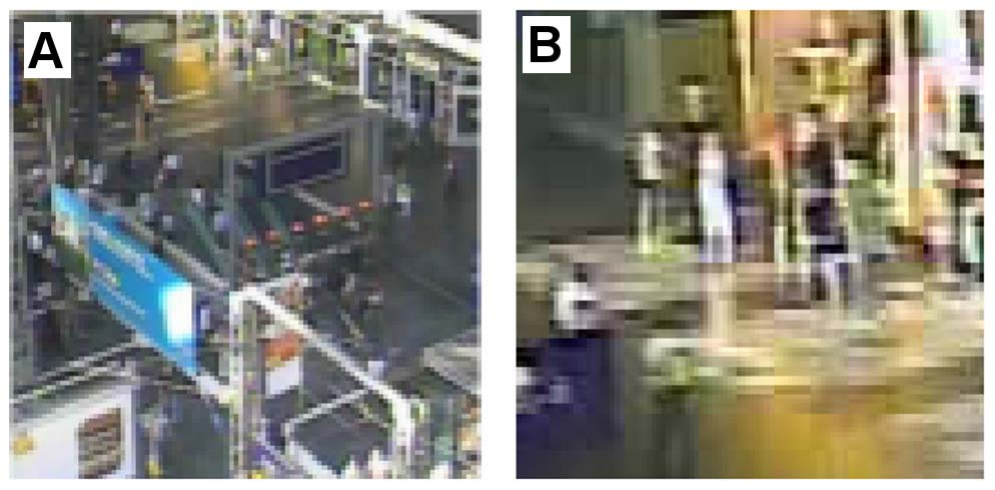
Snapshot of the observation area from one of the cameras. Left (A): Entire observation area: There are platforms on the left side, obstacles, such as food stalls, in the middle, and a wall on the right side. Right (B): Focus on moving pedestrians. For privacy's sake the second snapshot was blurred.

Our analysis of pedestrian motion is based on video recordings at the major German railway station. The video data was collected at the station in the morning (7 a.m.) and in the afternoon (between 4 p.m. and 6 p.m.) on a workday. The examined area covers several platforms and a part of the station's main hall. The video recordings show different scenarios at the railway station: A train departure and arrival and pedestrians entering the main hall and heading towards further destinations such as exits and food stalls. In all scenarios bi-directional or multi-directional flow was present. Figures 1 and 2 show a schematic picture of observation area highlighting the fact that one has to deal with hidden areas when using video recordings data.

**Figure 3:**
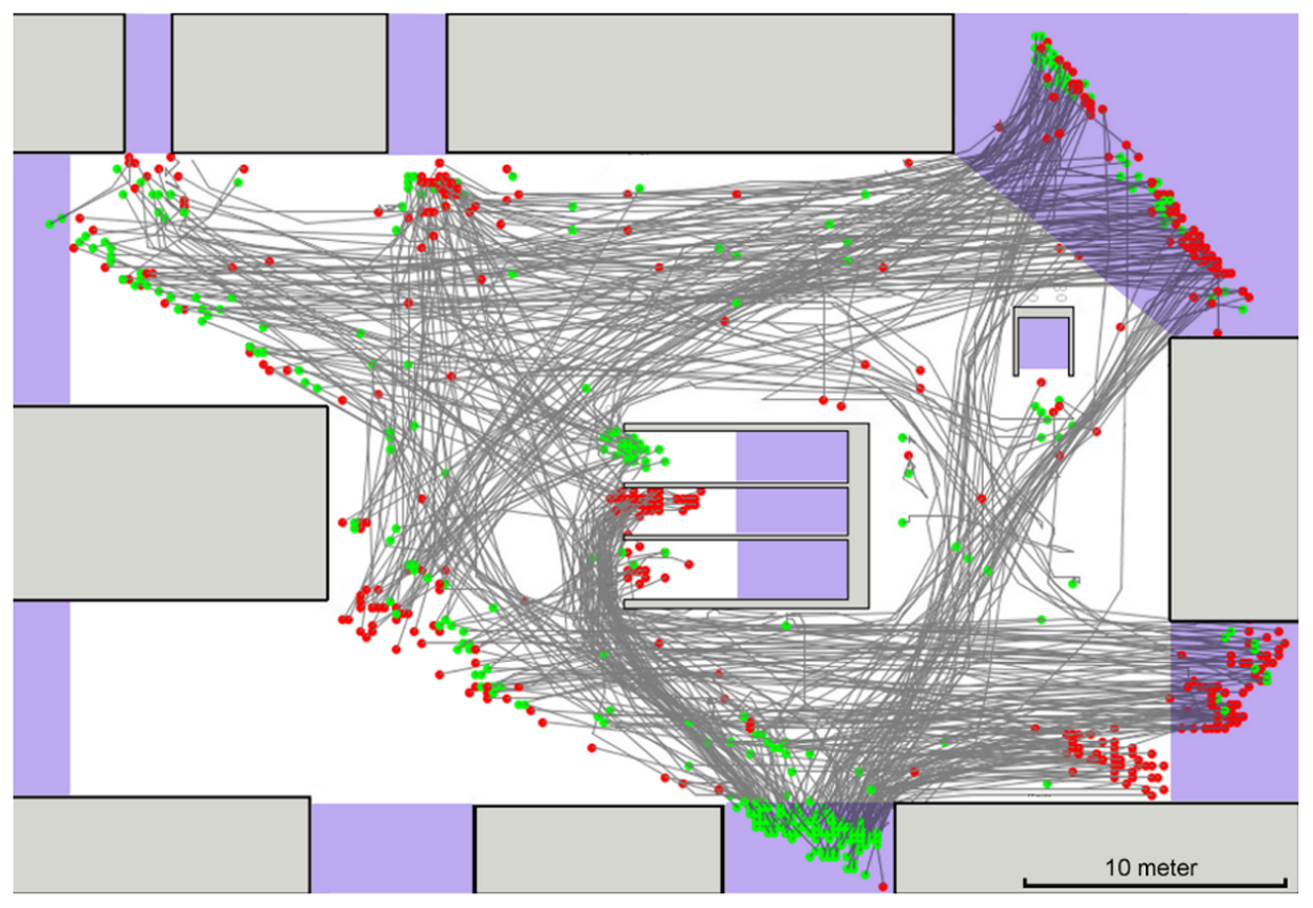
Example of a railway station scenario. Pedestrian trajectories (dark gray lines) were extracted from video footage. Green/red points correspond to the first/last detected pedestrian positions respectively. Light gray rectangles represent obstacles, lavender areas – possible entries and exits. Rectangles in the middle lead to escalators.

Trajectories of individual pedestrians in time and space were extracted from the video recordings and then analyzed. We used partly automated video tracking to extract pedestrian paths, walking speeds, schedules of pedestrians appearances and disappearances and pedestrian densities. Figure 3 shows trajectories extracted from one of the video recordings. The topology of the area of interest, the schedule of pedestrians appearances and disappearances and the path distributions are scenario-dependent. Walking speeds and flow-density dependencies have a general character and are analyzed in detail below.

#### Distributions of free-flow velocities

**Figure 4:**
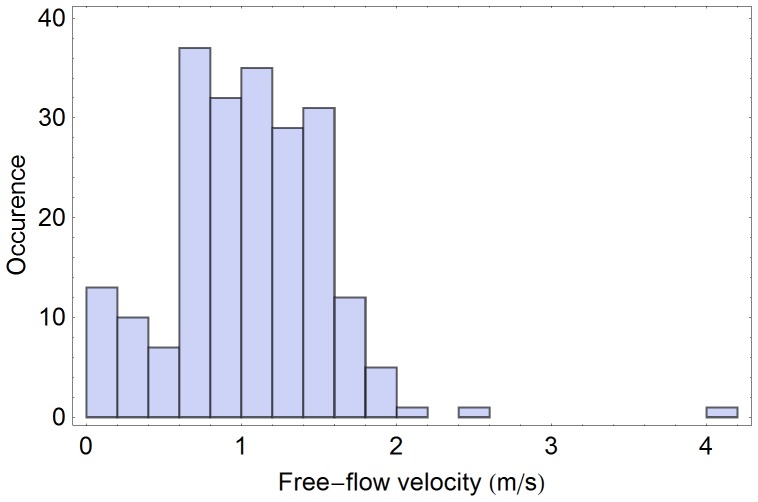
Histogram of free-flow velocities at 17:26 p.m. at a German railway station. All detected trajectories with free-path in the direction of movement are considered (214 samples). The mean free-flow velocity is 1.04 m/s and the standard deviation is 0.51 m/s.

**Figure 5:**
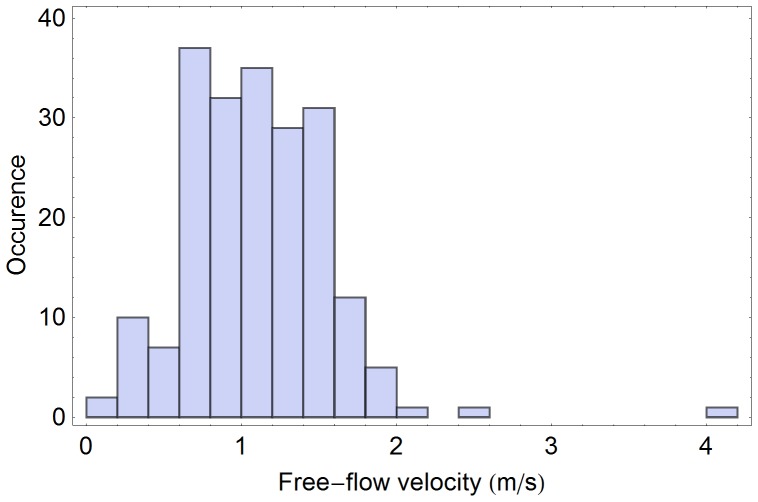
Histograms of free-flow velocities at 17:26 p.m. at a German railway station. Only trajectories with a free path in the direction of movement were considered. Obvious dawdlers with a velocity below 0.1/s and one runner with a velocity above 4.0 m/s were excluded as outliers. Thus 202 samples remained, resulting in the mean free-flow velocity of 1.08 m/s and the standard deviation – 0.42 m/s.

**Figure 6:**
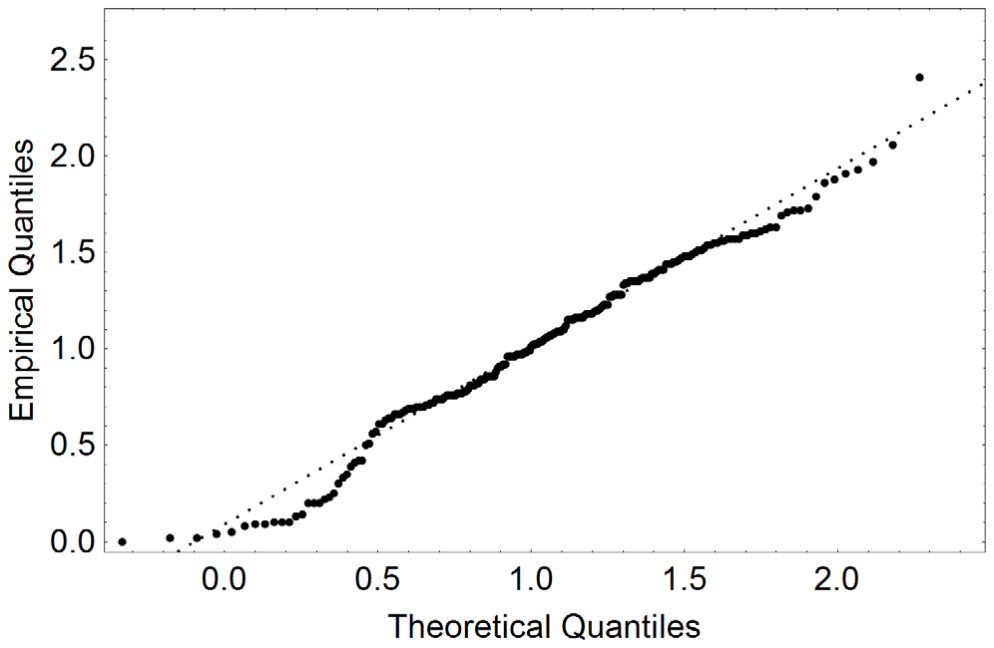
Quantile plots of free-flow velocities comparing the distribution measured at 17:26 p.m. at a German railway station to a normal distribution. All detected trajectories with free-path in the direction of movement are considered (214 samples).

**Figure 7:**
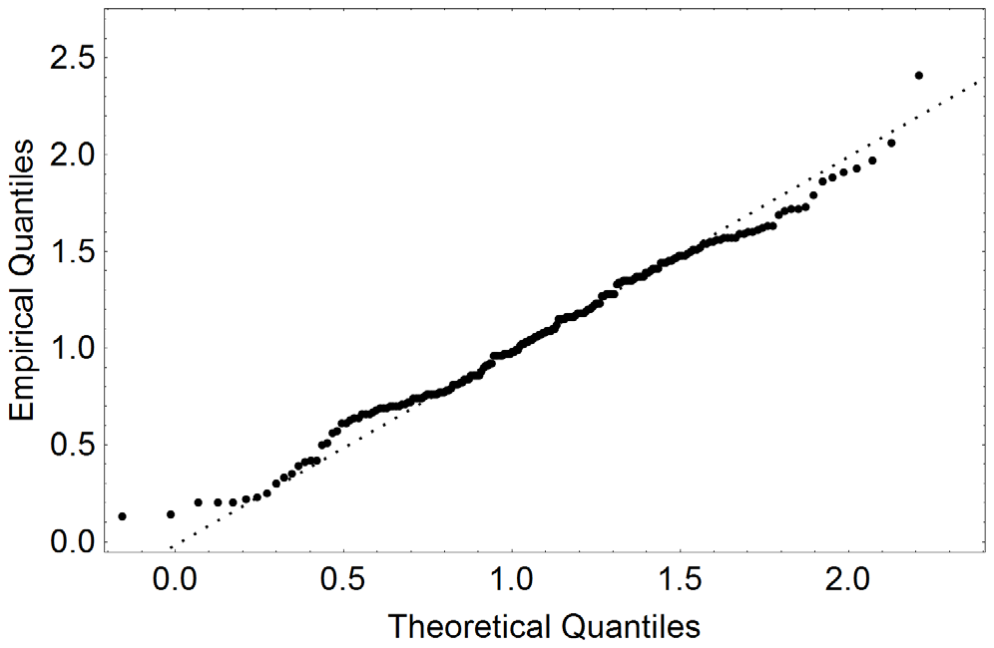
Quantile plots of free-flow velocities comparing the distribution measured at 17:26 p.m. at a German railway station to a normal distribution. Obvious dawdlers with a velocity below 0.1/s and one runner with a velocity above 4.0 m/s were also excluded. Thus 202 of 214 samples remained.

In literature it is mostly assumed that free-flow velocities are normally distributed [12]. With the availability of real-life measurements it makes sense to test the validity of this assumption: Here we test a null-hypothesis on normal distribution of free-flow velocities based on video analysis data (Figure 4, Figure 5). Our analysis shows that, the null hypothesis need not be rejected at the 0.5% level according to the Cramer-van-Mises test (Figure 6, Figure 7). Strictly speaking, assuming a normal distribution is wrong, because we neither allow negative speeds nor speeds above the current men's world record for sprints (which is slightly below 10 m/s). For the purpose of the simulation, however, the error seems to be acceptably small and we assume a normal distribution. Another assumption concerns loiterung and slowly moving people. Since it is difficult to distinguish between the two we exclude outliers in our benchmark simulation.

Weidmann claims that pedestrians on their way to work in the morning are 0.2 m/s slower than on their way home in the afternoon [12]. We observed a similar difference between morning and afternoon: In the morning the mean free-flow velocity was 0,97±0.1 m/s compared to 1,04±0.1 m/s in the afternoon. At the same time, even in the afternoon, our measurements showed a much lower mean free-flow velocity than 1.34 m/s generally assumed.

To be more precise, in the morning we observed a normal velocity distribution with the standard deviation of 0.29 m/s around the mean of 0.97 m/s. There was only one outlier (a passenger with a velocity below 0.1 m/s or above 4.0 m/s) among the 133 pedestrians with virtually no effect on the statistical outcome. In the afternoon the free-flow velocities were also normally distributed with the standard deviation of 0.51 m/s and the mean of 1,04 m/s. The situation remained unchanged after exclusion of outliers – 203 data points remained with the mean free-flow velocity of 1.10 m/s and the standard deviation of 0.47 m/s (see Figure 6). It should be noted that the outliers did not affect the overall dynamics neither in the morning nor in the afternoon as they we were only observed in situations with very low densities and therefore did not act as obstacles.

Compared to the benchmark data in [12] with the mean free-flow velocity of 1.34 m/s and a standard deviation of 0.26 m/s, the pedestrians observed at the railway station were slower and more diverse. We would like to point out that these observations may depend on the cultural background and that even qualitative results should not be carried over to other scenarios without verifying.

### Currently prevailing density-flow relationship: fundamental diagram

**Figure 8:**
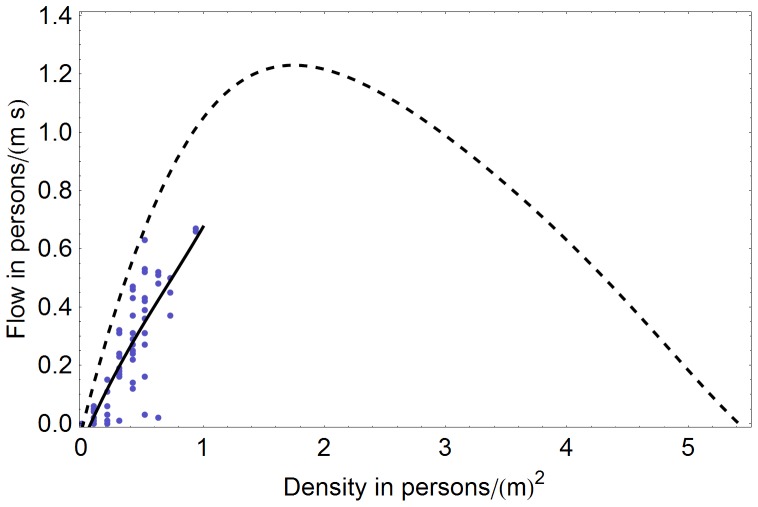
Measured density-flow relationship at a German railway station at 17:26 p.m. on a workday compared to Weidmann's diagram (dashed line). The maximum difference in flow between Weidmann's data (already given in a smooth form) and the measured data was 0.4 persons/ms. Densities above 1 person/m^2^ did not occur. The solid line is a smooth approximation of the measured data.

The density-flow relationship observed on the video records also deviated from the fundamental diagram provided by Weidmann which is usually associated with a mean free-flow velocity of 1.34 m/s (Figure 8). The maximum difference in flow was 0.4 persons/ms at a density of a little less than 1 persons/m^2^. Unfortunately higher densities did not occur in that scenario. 90% of the measured data points were within 0.15 persons/ms of the smooth approximation of the data.

### Simulation tool

#### Pedestrian stream models

There are various models of pedestrian motion, all of them with their own merits [17], [22], [26]–[34]. On www.ped-net.org alone, 65 different tools are listed, not including the tool described here. For more complete surveys we refer to [35]–[39] with descriptions of a large number of approaches [9], [36], [40]–[46] for modeling pedestrian movements.

Two particularly well established classes of pedestrian motion models, cellular automaton based models [47]–[50] and social force models, [22] both embrace similar ideas: Both use inspiration from similar physical models, such as the idea of repulsive forces between pedestrians to ensure that they keep a distance among each other. The mathematical formulations, however, are very different. Despite the differences, the methodology presented further is suitable for both classes of models and for any other model that is able to calibrate according to the parameters and measurements we describe below, as for example, [51]–[53].

### Cellular automaton model

In this section we describe our benchmark simulation tool. It has been introduced by the authors and by their colleagues in earlier publications [54]–[56]. Hence, we restrict this description to the minimum necessary to understand the paper. Although the methodology we present here is independent of the specific model, comprehending the main principles of our model helps to identify how empirical results may serve as input. In particular, we aim to identify the data that can be used directly as input from observations and the data that serves as a basis for model calibration.

Our goal is to construct a simulation model that is capable of reproducing real-life scenarios and preemptively predicting critical situations, such as life threatening local densities, faster than real-time. That is, it must build on observational data and allow for exceptional computational speed. Our benchmark simulation tool based on a cellular automaton fulfills both requirements.

Since we stick to measurable data, many psychological aspects are neglected in our model. Differences between virtual pedestrians are extremely reduced, and the pedestrian model is based on very few simple assumptions:

- Pedestrians “know” the shortest path. Limited vision of pedestrians is neglected as well as incomplete knowledge of the terrain. Pedestrians move from their current positions towards individual targets along shortest obstacle-free path, unless such a path is blocked by a fellow pedestrian.
- They move at individual preferred speeds – the free-flow velocities – as long as the path is free.
- Each individual has a need for private space that depends on his/her current situation. This need is expressed in the distances that individuals try to keep from each other. It also makes people keep distances from obstacles such as walls.
- Pedestrians decelerate when the local density in their direction of movement is increased.
- All further individuality, such as age or fitness, is captured by the personal free-flow velocity.

Clearly, the behavior of pedestrians is strongly simplified with this approach. This has two major advantages: The number of input parameters that must be procured from measurements is low whereas the computational speed of the simulation tool is very high. Both aspects are of extreme practical importance.

#### Cellular automaton

**Figure 9:**
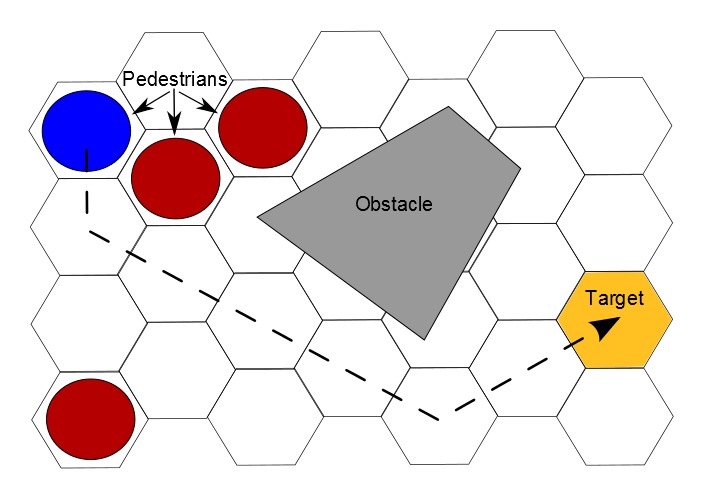
Cellular automaton model: Pedestrians move towards a target on hexagonal cell grid. Persons, targets and obstacles occupy cells. Positions are updated sequentially in each simulation step so that collision is impossible.

As in any cellular automaton model, the area of observation is divided into a lattice of cells (Figure 9). Although square cells seem to be the most popular choice, we prefer a hexagonal grid for its two additional natural directions of movement compared to the square grid. The cell diameter is set to 53 cm to accommodate an average sized Caucasian male. This parameter can be adapted to better fit e.g. Asian pedestrians or children. Each cell at each time step has one of the following states: either empty or occupied by either a person, an obstacle or a target.

#### Spatial dimensions and topology

Pedestrians move on a single plane or on multiple planes such as floors. This allows us to consider two spatial dimensions only. Virtual persons enter and leave the scenario through sources and targets, namely entrances and exits. Sources and targets have three types of parameters – their positions, schedules of pedestrian generation/disappearence and, for the sources, also source-target distributions, that give probabilities for selecting pedestrian destinations when generating pedestrians. All these parameters can be taken directly from measurements and fed into the simulation.

#### Potential fields

In many aspects, our model is similar to other cellular automaton models based on potentials [43], [48], [57]–[59]. In particular, we borrow the inspiration for the rules from electrostatics: Pedestrians are treated as negatively charged particles, say electrons. Pedestrians are attracted by positive charges, such as exits, and repelled by negative charges such, as other pedestrians or obstacles. The forces between pedestrians, targets and obstacles are expressed through suitable scalar functions, the potentials, which are summed up to form an overall potential field. We choose the potential described in the following, note, however, that different approaches could be used for constructing the potential fields [38], [60].

- Each virtual person carries around his or her own potential given by a radially symmetric function of type 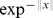 that is almost zero a few cells away from the person.
- Obstacles like walls are assigned positive potentials to make “people” prefer to keep a distance.
- The long–range attracting potential of a target is coded in a floor field that corresponds to the arrival time of a wave front traveling with constant speed from the target through the space formed by the obstacles and boundaries of the scenario [55]. This ensures that each pedestrian moves along the shortest path to his or her target as long as this path is free from other pedestrians.
- It is also possible to include clumps of pedestrians in the way to a target in the computation of the floor field thus adding a further dynamic aspect to the potential [55].

When a virtual person steps ahead he or she selects the empty neighbor cell with the steepest descent of the overall potential, thereby obstacles and other persons are successfully skirted. The repulsive potential of a fellow pedestrian on the shortest path leads to either evasion or slowing down.

#### Sequential update scheme

Simulation dynamics follows a specific kind of sequential update scheme. Each person has an individual speed, the free-flow velocity, which he/she tries to achieve – and indeed does achieve when the path is free. The value of the free-flow velocity prescribes how often the corresponding person will be chosen for an update: At each time step all persons are identified that are allowed to move. Faster persons are chosen more often so that on average each person moves with their prescribed speed as long as the path is free. The positions of the chosen pedestrians are updated in the order of their “life-time” in the simulation – that is, the time that has elapsed since their generation.

Using the terminology in [57] our model is: microscopic, discrete and deterministic with stochastic aspects, rule-based but potential-driven.

### Methodology

This section describes our methodology of comprehensive calibration against a real-life scenario. The first step towards calibration is to identify the key parameters that must be input into the simulation program. In the previous section we have described the data required for our benchmark model: positions of sources and targets, shapes and positions of obstacles, a pedestrian appearance schedule, source-target distributions, velocity distributions and flow-density dependency. Some of the parameters, such as the location and form of obstacles, can be extracted from the data sources and fed directly into the simulation; Other parameters must be estimated by statistically analyzing the data. Some input even comes in the form of a function such as the density-velocity relation.

Most parameters change with time and must be calibrated at suitable intervals. Here, we distinguish between quasi-stationary and dynamic parameters: Dynamic parameters change quickly and have an immediate strong impact on the prediction; Quasi-stationary parameters must be re-calibrated at regular intervals of several prediction periods (whereas stationary input needs to be adapted on demand only). In general, what inputs are stationary, quasi-stationary or dynamic depends on the scenario. Distinguishing among different parameter types is important since it helps to determine how often one should adapt the simulation with respect to a certain type of parameter. In the following we describe our classification which appears suitable for our railway station scenario. Still, the principles listed below have a general character and can be applied to various scenarios.

The typical duration of a simulated railway station scenario is several minutes and the classification introduced further is related to this duration. We suggest constructing a “basic scenario” from the observations, and then calibrating the more volatile information in shorter intervals:

- *Stationary input* – stationary data can be expected not to change over a period of time significantly longer than the scenario duration. For a railway station, the input is assumed to be stationary if it does not change in a range from several hours, to days, weeks, or even months, etc., – for example, the locations of permanent structures such as platforms. However, even this type of data must be checked in regular and relatively short intervals because any alterations in the infrastructure may dramatically impact the scenario.
- *Topology* – the area of observation could be divided into walkable and non-walkable areas. The latter includes obstacles with their types, positions and forms and areas where pedestrians are not allowed to enter, such as railway tracks. This data is fed directly into the simulation and can be assumed to remain stationary.
- *Positions of sources and targets* – the topology also determines possible positions of sources and targets for virtual pedestrians, that is, the locations where people come from and where they go to. For a railway station these can be: Departure and arrival platforms, entrances and exists. Again, this is direct input data that is mostly stationary. One should, however, check this information at some regular intervals since entries and exits can get closed at any time.

#### Quasi-stationary input

By quasi-stationary parameters we mean parameters which gradually change over time but can be considered stationary for several prediction periods. On the scale of our simulation scenario, they can be considered stationary for a few minutes. Such parameters often depend on the time of day or type of scenario. We recommend to store typical values in a scenario data base and to use them as default starting values for fast and yet accurate calibration:

- The *Distribution of free-flow velocities* is very often taken from literature following Weidmann's suggestions [12] of a normal distribution with the mean velocity 1,34 m/s and the standard deviation of 0,26 m/s. In *Distributions of free-flow velocities* we described how the results of our free-flow velocity measurements differ from literature values (Figure 4, Figure 5). Therefore, in contrast to the usually assumed distribution, we propose to extract the distribution of the free-flow velocities from observations and to generate virtual pedestrian velocities according to the empiric distribution from the last measurement. Note, if a scenario contains stairs, escalators or other types of non-standard planes, the free-flow velocities should be measured additionally for these surfaces. For example, for stairs and escalators up and down the free-flow velocity should be measured separately.
- *Density-flow relation* – often Weidmann's diagram is used as fundamental diagram to describe the density-flow relation. However, our measurements suggest that the real behavior of pedestrians may differ (Figure 8). Therefore, we propose to use a measured flow-density relation instead. In particular, to deal with measured scattered values, we propose to use a smooth approximation or piecewise interpolation of the measured density-flow relation as a reference curve, or objective function, for calibration. This can be achieved robustly with standard optimization methods [54], [61]. We expect the relationship to be quasi-stationary which is a very desirable quality to speed up the calibration process: The last set of calibrated parameters gives an excellent starting position for the next calibration; thus, calibration time would not become an issue. However, the assumption of quasi-stationarity of the density-flow relationship remains to be further substantiated through more extensive measurements. Note that similar to the case of the free-flow velocity, the density-flow relationship should be measured separately for stairs, escalators, etc.
- *Distance kept from walls* – when moving, pedestrians keep a certain distance from obstacles. In [62] movement of a single person in the presence of walls is examined. We propose to investigate multiple trajectories to derive a distribution of the distances kept to the walls. This distribution can be taken as a reference curve according to which the simulation tool can be calibrated. The statistical data on the distances to walls in our video footage is under investigation at the moment. First observations indicate that the influence range of a wall is about 2 m.
- *Source-target relationship* – to direct virtual pedestrians from sources to targets in a way that fits the scenario, statistical information on mapping between sources and targets must be extracted from video footage, or any other type of suitable sensor. The analysis of the video data for our railway station suggests that the distribution differs from scenario to scenario and depends on time, however, usually it changes on time intervals larger than the simulated period of time. Hence we propose to re-evaluate the target-source distribution at relatively short intervals to direct virtual pedestrians from sources to targets in a way that fits the scenario.

#### Dynamic input

Dynamic parameters change very quickly and therefore demand constant recalibration. An obvious example are pedestrian appearances following, e.g. train arrivals. Neither arrival times nor train occupancies are predictable with precision, so pedestrian appearances are volatile. Since this kind of information changes every few minutes, it limits the maximum span of predictions intervals accordingly. A permanent readjustment process is necessary here.

*Schedule of pedestrian appearances and disappearances* – how many pedestrians per second appear from a source? How many disappear at a sink or a target? This data is necessary to feed virtual pedestrians into the simulation and remove them in accordance with the scenario. At the same time this schedule is very volatile.

### Proof of concept: validation and sensitivity study

In this section we describe the validation of the model by comparing simulation results to measurements. We also present the results of a sensitivity analysis conducted to determine the parameters, whose small changes have a significant impact on the simulation results. For sensitive parameters, accurate measurements are critical if one aims at reproducing real-life scenario and performing predictive simulations.

#### Validation goal

The goal of validation is to make certain that a model used to describe some complex real system well matches the characteristics of this system. Our benchmark model was first validated with respect to a number of phenomena and data from laboratory experiments following the suggestions for tests of the RiMEA validation initiative [63]. Then we conducted our own validation tests described in [61] and [64]. In this section we describe the validation of our benchmark model for the real-life railway station scenario.

#### Quantities suitable for comparison

The simplest way to validate a model against real-life data is to compare video recordings with simulation visually. This is a valuable plausibility check to which validation has been largely restricted [9], [65] so far. As far as quantitative validation has been attempted in previous investigations, it was performed based on trajectories, flow or velocity comparisons [24]. Here, we would like to go further and identify quantities suitable for forecasting critical situations such as critical densities. For this, we pick the crowd density as it evolves with time in the area of observation: It can be measured quite easily in both, real scenario and its virtual reenactment and is of high practical interest since local high densities indicate *hot spots* where the risk for accidents is elevated.

#### Comparison of the real-life scenario with the virtual reenactment

As soon as adjustments and calibration against measured data have been performed, a predictive simulation can be started. The velocity distribution, the density-flow relation, the schedule of appearances and disappearances of pedestrians, the source-target distribution and the positions and shapes of sources and targets (s. *Methodology*) were extracted from video recordings and used as input.

**Figure 10:**
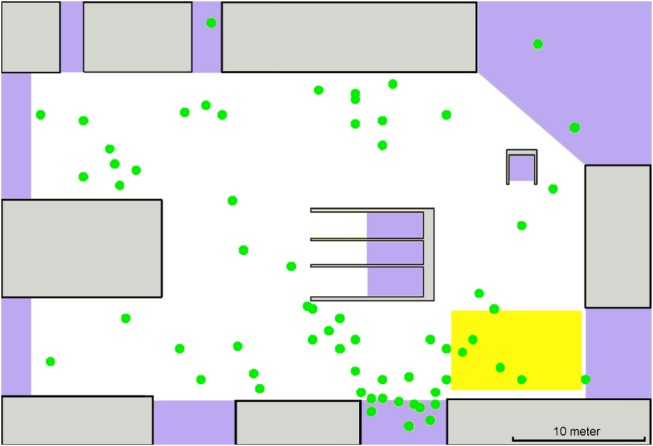
Snapshot of a simulated scenario. Circles correspond to pedestrians. Lines give contours of obstacles, sources and targets locations. The rectangle covered by a grid (lower left corner) shows the area of observation.

For the model validation we choose the most challenging scenario (see Figure 10) where the highest densities together with multi-directional flow were observed: A train arrives, passengers disembark and walk towards exits. There are also other pedestrians walking in the main hall. At first, the pedestrian density is relatively low until the bulk of passengers passes through the area of observation. Then the density decreases again until all passengers from that train are gone.

**Figure 11:**
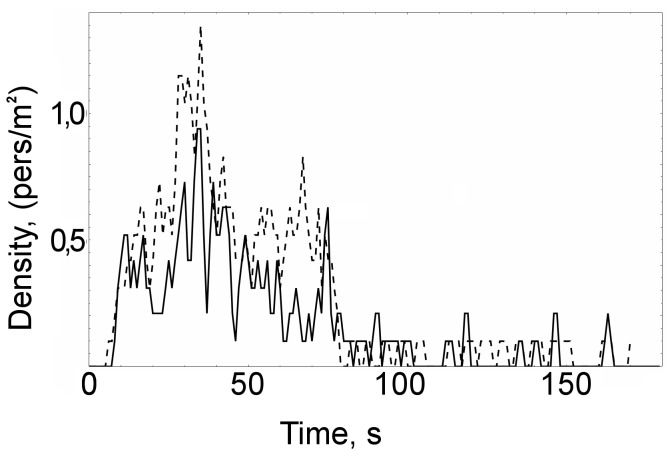
Comparison of densities measured on video footage (solid line) to simulated densities (dashed lines) on the area of observation shown in Fig. 10. Pedestrians are fed into the scenario with the source target distribution, free-flow velocities and the density-flow relationship measured at the start of the scenario. 180 seconds (3 minutes) are simulated.

To compare the simulation and the data extracted from the video recordings let us first look at the density evolution in the most busy and complicated area in the scenario: the rectangle covered by a grid as shown in Figure 10. Figure 11 shows the comparison of densities (simulated to measured) in a time span of 3 minutes: The solid line corresponds to the video footage, the dashed line to the simulation.

Consider Figure 11: The simulation reproduces the scenario quite well: The pedestrian density peak occurs at the proper area and time, and it has the correct duration and order of magnitude. However, the simulation somewhat overestimates the density. Part of the difference can be explained by the influence of chance: The simulation is subject to random input. For each new seed the results differ so that individual velocities and trajectories cannot be expected to match. Only a statistical match of measurements and simulation results is possible.

We also suspect that real pedestrians coordinate their movements better than virtual pedestrians do: Our virtual pedestrians are quite “short-sighted” and take steps to avoid collision only when they actually “feel” the potential of other pedestrians; Real pedestrians are more likely to plan ahead. This is a typical disadvantage of so-called greedy algorithms that rely on locally optimal choices to enable high-speed simulations.

The important question is whether such systematic overestimation is acceptable. In our case we are interested in a warning system for potentially dangerous densities. Therefore we believe that a slight overestimation can be tolerated, whereas any underestimation would render the model unusable.

**Figure 12:**
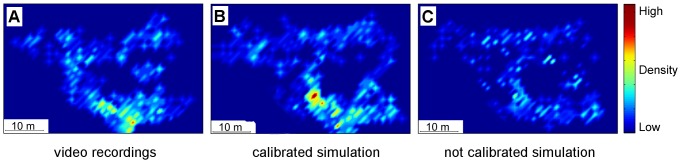
Comparison of pedestrian densities extracted from the video recordings with the predictive simulation. Densities are averaged over 6 seconds. Snapshot is taken 40 seconds after a train arrival: at time when pedestrian density peak occured.

So far the comparison was restricted to a single rectangle in the area of observation. As a next step we look at the whole area of observation. The results are represented in Figure 12. We observe that the density magnitudes are very similar for both, the video recordings and the calibrated simulation, in the whole area of observation. The slight density overestimation at the hot spot is also visible (Figure 12, calibrated simulation). Most importantly, the position of the density peak in the simulation is very close to the one in the video recordings. In contrast to that, the uncalibrated simulation is not capable of reproducing the observed data. No high densities occur and the small spikes appear randomly.

#### Sensitivity analysis

We finally investigate how the uncertainty in our model input affects its output: For which parameters does a small change cause a significant change in the simulation results? These sensitive parameters should be measured very carefully whereas non-sensitive ones require only a rough estimation.

We introduce a target function, the *fitness*, that quantifies the precision of our simulation. The higher the fitness the better the match between observation and simulation is supposed to be. To calculate the fitness, we cover the scenario area with *N* measurement tiles of unit size 1 m×1 m. In each tile we measure the distance between the observed and simulated densities by counting the number of pedestrians in each unit tile every two seconds (the two second interval is chosen in accordance with the observed velocities). The fitness at time *t* is given by the inverse of the sum over all measurement tiles: 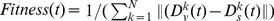
(1)


Here, *N* is the number of tiles and 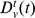 and 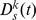 are the pedestrian densities observed on the video and in the simulation at tile *k* at sample time *t*. Overall fitness is obtained by summing over all samples in time. This approach quantifies how closely the local densities in the video recordings and in the simulation evolve. The calibrated parameters gathered when applying our methodology yield the benchmark fitness.

**Figure 13:**
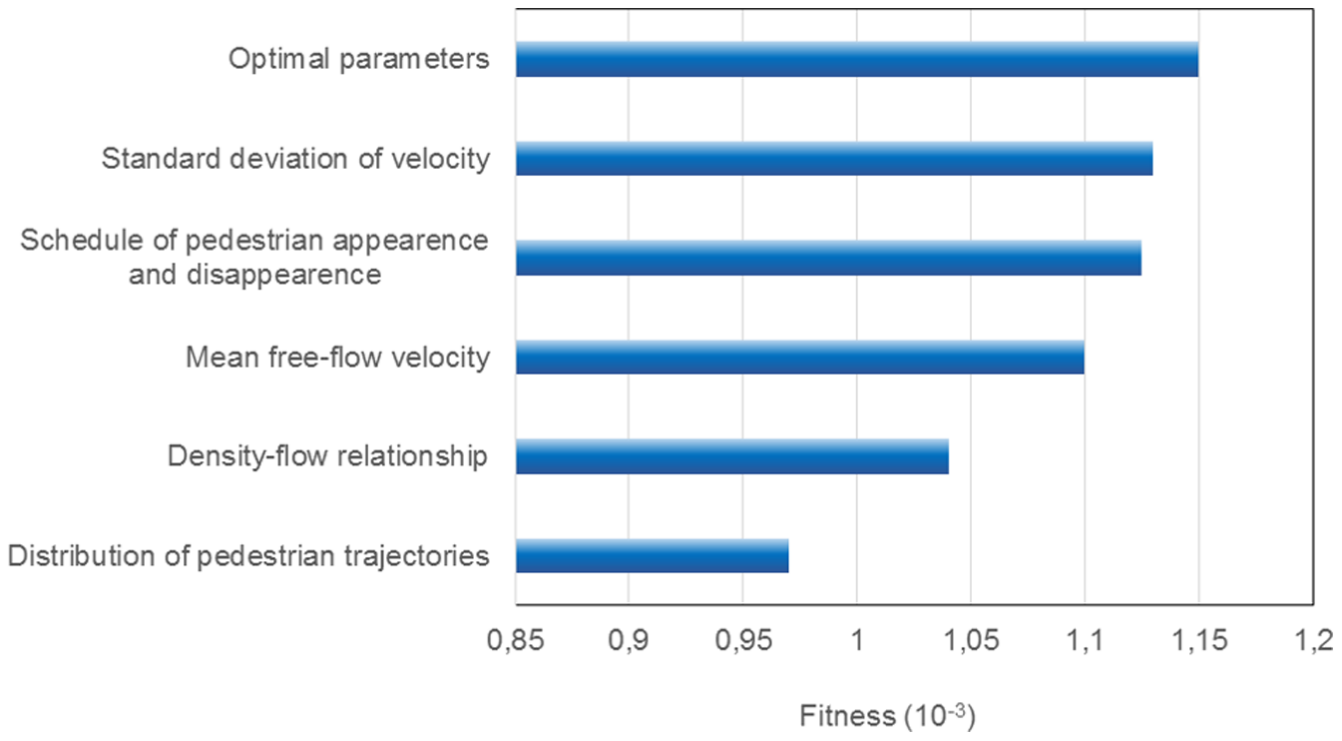
Sensitivity analysis of parameters. The influence of parameter deviation on fitness, i.e. accuracy of simulation, is evaluated. The upper bar “Optimal parameters” corresponds to the fitness reached with parameters gathered by applying the proposed methodology. Other bars show how fitness changes if a parameter value is deviated 10% from optimal value.

To conduct the sensitivity analysis, we compare the fitness of the simulation outcome when varying the parameter values by 10%. Figure 13 illustrates the influence of the crucial parameters on the quality of the match. The source-target distribution seems to be the most sensitive parameter and should be measured very precisely for a scenario. Also the schedule of pedestrian appearances on the scenario and the velocity distribution play a very important role for the predictive power of the simulation.

## Discussion and Conclusion

Pedestrian stream simulations are only helpful for gaining virtual experience and for real-time prediction when they are able to reproduce the corresponding real-life scenario. A vital step to achieve this is calibration and validation of a model against the real-life scenario. So far, the data on realistic behavior of pedestrians under natural conditions was very limited [66]. Calibration and validation in many models was mostly performed for an isolated phenomenon observed in a controlled experiment or for a relative small observation area in case of real-life scenarios as in [22] etc. Therefore, the question was open: How to calibrate and validate a pedestrian stream simulation against complex real-life scenarios?

To answer this question we addressed a number of subquestions:

- Are standard values for free-flow velocities and density-flow relations always valid?
- What characteristics of pedestrian dynamics should be extracted from real-life scenario for simulation calibration?
- Which characteristics have a high impact on simulation accuracy?
- How to calibrate a simulation based on characteristics of pedestrian dynamics extracted from video recordings?
- How to validate a model quantitatively after calibration?

To answer these questions we first analyzed video recordings of a real-life scenario at a major German railway station. We provided experimental evidence that people at the railway station walk significantly slower than standard literature suggests [12]. These results support the idea that free-flow velocities depend on the environment [22]. Pedestrians also decelerate more strongly than Weidmann′s fundamental diagram indicates [12]. This underlines the importance of scenario-specific measurements as input data and calibration to measured data, especially if predictive simulations are attempted.

Based on data extracted from video recordings we suggested and applied a methodology for model calibration against our real-life scenario. The benchmark model was based on a cellular automaton. The most important aspects and characteristics taken into account are: scenario topology, sources and targets positions, statistical distribution of trajectories between sources and targets, schedule of pedestrian appearances and disappearances (in the scenario), distribution of free-flow velocities, prevailing density-flow relationship, and distances that pedestrians keep from obstacles. Some of the parameters are direct input parameters while others, like the density-flow relationship, are expressed through target functions for parameter adjustments.

We demonstrated that the proposed method significantly improves the quality of simulation and the potential prediction accuracy: The success of the proposed approach was tested by comparing evolutions of simulated and observed pedestrian densities. The simulation predicts the density evolution correctly, both qualitatively and quantitatively: Maximum density is somewhat overestimated but never underestimated, so that the simulation can be used as a predictive warning system for potentially dangerous local densities. We also found that the most sensitive parameters are the source-target distribution, the pedestrian appearance schedule and the free-flow velocities. These core parameters should be measured with high precision as they strongly influence the accuracy of the pedestrian simulations.

The proposed method is a first step towards comprehensive calibration and adjustment of simulations to complex real-life scenarios. The proof of concept with a benchmark simulation tool shows that short term predictive simulations are possible as long as the crowd is sufficiently dense to allow statistical interpretation. This also defines the limits of pedestrian simulation: Individual behavior can only be captured and reproduced in a statistical sense. Dangers that are triggered by highly individual behavior may be investigated as far as their effect on the crowd is concerned, but one cannot predict when they occur or whether they will occur at all. Furthermore, fast changes – sometimes within seconds – of very sensitive input data, such as pedestrians appearance, make clear that longer-term forecasts can only be understood as possible outcomes among other conceivable scenarios. The only useful procedure here is to simulate scenarios and observe possible outcomes to gain virtual experience.

Hence, longer term simulations can be seen as valuable contributions to risk analysis, while short-term simulations have the potential to support immediate decisions on safety issues. For example, a short-term prediction could help to decide whether train passengers should be allowed to disembark when a train station is already crowded.

Further steps to improve the method can include considering group behavior: In our benchmark scenario single commuters dominated the crowd so that there was no need to incorporate group behavior as in [56] and [67]. However, if groups are present in a crowd, their effect cannot be neglected [68].

## Materials and Methods

### Data extraction from videos

All trajectories were extracted from the video recordings using a partly automated tool that allowed to “click” positions on the video recordings. We analyzed five recordings in total, each of which had a duration of at least 1.5 minutes. The number of pedestrians on each recording was about 400. One video recording was made at 7 a.m. in the morning of a workday, other recordings were made in the afternoon between 4 p.m. and 6 p.m., also on a workday.

For each trajectory detected on video recordings, its source and its target were identified: usually the first and the last detectable position. Some meaningful sources and targets may have been obscured by obstacles or hardly visible from the camera angle. In such cases we used additional information like the direction of pedestrian movement to identify, for example, a likely exit. Based on this information we identified how likely a person coming from source A is to choose target B thus collecting the source-target statistics. Also using the extracted trajectories resolved in time, we were able to derive the schedule of pedestrian appearance at each source and disappearance at each target on the scenario.

Trajectories that were partially obscured from the camera view or where the view was distorted by distance were only used to gather source-target statistics. Detailed analyses of velocities, flows and distances to walls were conducted exclusively on the visible and undistorted parts of the trajectories to keep measurement errors small.

While the programs we used also introduced errors, we estimate that they were small compared to the error committed by manually pinning down the center position of each person's head when tracking pedestrian trajectories. We had no cost-effective way to systematically investigate this error. However, we think it is safe to assume that the head was always correctly identified, but with a deviation from the center. In this case, an error in position would not exceed 9 cm – the radius of a circular approximation of a human head.

### Ethics

No ethics statement is required for this work. The video footage was recorded by the train station operator, Die Bahn, in accordance with § 6b (1) of the German Data Protection Act (DPA) and provided to us already anonymized (in very low resolution making no personal identification possible, § 3 (6) of the DPA). Usage of such anonymized data is exempt from further regulation under DPA as per § 1 (2).
